# WGCNA Analysis Identifies the Hub Genes Related to Heat Stress in Seedling of Rice (*Oryza sativa* L.)

**DOI:** 10.3390/genes13061020

**Published:** 2022-06-06

**Authors:** Yubo Wang, Yingfeng Wang, Xiong Liu, Jieqiang Zhou, Huabing Deng, Guilian Zhang, Yunhua Xiao, Wenbang Tang

**Affiliations:** 1 College of Agronomy, Hunan Agricultural University, Changsha 410128, China; wangyulive@163.com (Y.W.); wangyingfeng229@163.com (Y.W.); xiongliu@whu.edu.cn (X.L.); crackzjq@163.com (J.Z.); denghuabing@126.com (H.D.); zgl604@163.com (G.Z.); 2 State Key Laboratory of Hybrid Rice, Hunan Hybrid Rice Research Center, Changsha 410125, China

**Keywords:** WGCNA, heat stress, modules

## Abstract

Frequent high temperature weather affects the growth and development of rice, resulting in the decline of seed–setting rate, deterioration of rice quality and reduction of yield. Although some high temperature tolerance genes have been cloned, there is still little success in solving the effects of high temperature stress in rice (Oryza sativa L.). Based on the transcriptional data of seven time points, the weighted correlation network analysis (WGCNA) method was used to construct a co–expression network of differentially expressed genes (DEGs) between the rice genotypes IR64 (tolerant to heat stress) and Koshihikari (susceptible to heat stress). There were four modules in both genotypes that were highly correlated with the time points after heat stress in the seedling. We further identified candidate hub genes through clustering and analysis of protein interaction network with known–core genes. The results showed that the ribosome and protein processing in the endoplasmic reticulum were the common pathways in response to heat stress between the two genotypes. The changes of starch and sucrose metabolism and the biosynthesis of secondary metabolites pathways are possible reasons for the sensitivity to heat stress for Koshihikari. Our findings provide an important reference for the understanding of high temperature response mechanisms and the cultivation of high temperature resistant materials.

## 1. Introduction

Global warming has become one of the serious challenges that mankind cannot ignore. High temperature hazards caused by warming have severely affected crop production and food security. It is estimated that for every 1 °C increase in the average daily temperature, yield losses in rice, maize and wheat will increase by 10–25% [1].

Heat stress (HS) is defined as irreversible damage to plants due to the temperature beyond a physiology threshold level [2]. Rice is a plant that is vulnerable to HS. HS affects the growth and development of rice at any time during the entire growth period, such as slowed growth rate, increased water loss, abnormal plant height and biomass at the seedling stage, changed grain shape, deterioration in rice quality and reduction in yield at the reproductive stage [3,4]. The physiological effects of HS on rice mainly include membrane damage, reactive oxygen species (ROS) accumulation, photosynthesis damage, disturbance of carbohydrate metabolism and partitioning, and phytohormone imbalance [5]. The molecular mechanisms of plant response to HS include remodeling of the cell wall structure, changes in cell membrane fluidity and Ca^2+^ concentration mediated by membrane localized Ca^2+^ channels [6,7]. To date, a number of thermotolerance genes and quantitative trait loci (QTL) have been discovered using the phenotypes induced by HS, such as seedling root, survival rates, yield per plant and seed–setting rates. However, only a few causal genes have been cloned, successfully validated in terms of their function and used for breeding, independent of genetic background [8].

With the development of data analysis methods and the further cost reduction of high throughput sequencing, RNA sequencing (RNA–seq) has become a routine technique in response to the various biotic and abiotic stresses of rice. The amount of HS, cold, cadmium, *Sogatella furcifera* and *Nilaparvata lugens* responsive genes and specific regulation pathways have been found in rice by omic data [9,10,11]. The processes of responding to various stresses often involved the interaction of multiple genes and different metabolism pathways. The conduction of RNA–seq involves the exploration of thousands of differentially expressed genes (DEGs) and systematically interpreting their biological functional characteristics [12]. The classic biological research method focuses on DEGs between the group pairs. Through a simple comparison of the biological functions, a few genes were selected for further research that utilized the single–dimension research method [13]. When comparing a large number of DEGs between multiple groups, gene co–expression networks have more advantages than traditional methods [14,15]. Gene co–expression network analysis, as one of the types of molecular biological networks, is a network graph constructed based on the similarity of the expression data between DEGs [16]. Each node is defined as a gene, the genes with common expressions in different samples are in the same gene network, and the co–expression relationship between the genes is generally measured by the expression correlation coefficient between them, so as to understand the interaction relationship between genes, find core genes and predict unknown gene functions [17,18].

Weighted correlation network analysis (WGCNA) is a system biology method used to describe gene association patterns between different samples. It can identify gene sets (modules) with similar expression patterns, analyze the relationship between modules and sample phenotypes, draw the regulatory networks between genes in the modules and identify key regulatory genes [19,20]. The WGCNA algorithm first assumes that the gene network obeys a scale free distribution, and defines the gene co–expression correlation matrix and the adjacency function formed by the gene network, then calculates the dissimilarity coefficients of different nodes and constructs a hierarchical clustering tree accordingly [21]. The different branches of the clustering tree represent different modules; the module genes have a high degree of co–expression, while the genes belonging to different modules have a low degree of co–expression [22]. Through exploring the relationship between modules and specific phenotypes, the identification of gene networks can finally be achieved [23].

Here, we used the *indica* variety IR64 and the *japonica* variety Koshihikari as study materials. IR64 is the parent of modern indica rice varieties. Koshihikari is an elite Japanese variety. Both IR64 and Koshihikari are the most popular *indica* and *japonica* rice varieties in the world and are widely grown in various rice producing areas. The transcriptome information of the two genotypes at seven time points after HS was obtained using RNA–seq. Based on these data, we performed WGCNA analysis to explore the highly correlated modules and co–expressed genes. By analyzing the interaction network between the co–expressed genes of the two genotypes and the studies of HS–related genes, the ultimate goal was to identify the hub genes and preliminarily explain the reasons for the different responses of two genotypes to HS.

## 2. Materials and Methods

### 2.1. Rice Varieties and HS

The moderately resistant variety “IR64” and highly susceptible variety “Koshihikari” were provided by the College of Agronomy at Hunan Agricultural University. Rice seedling plants were grown hydroponically in a growth phytotron at 28 °C with a 12 h day/12 h night cycle, and a humidity of 70% to 10 day old stage, then treated with 45 °C for HS.

### 2.2. Acquisition and Standardization of Transcriptome Data

Leaf samples were harvested at seven time points (0, 0.5, 1, 2, 4, 8 and 24 h) after HS, and 42 sets of RNA–seq data (two cultivars and three biological replicates) were obtained in this study. Both genotypes were based on 0 h (control), and a large number of DEGs were obtained at each time point (Supplementary Table S1A,B). The raw read counts of each gene were calculated, standardized and compared through the HTseq (1.99.2) and DESeq (1.34.0) functions of R.

### 2.3. Construction of WGCNA Co–Expression Network

The construction of the WGCNA co–expression network was achieved using the WGCNA package in R version 4.1.1. The average expression of each gene at different time points was calculated, and the genes with no change in expression were filtered out. The expression level of each gene was normalized 0–1, and Pearson’s coefficient was used to calculate the correlation between genes to determine the co–expression similarities of two genes. An appropriate adjacency matrix weight parameter β value was selected to satisfy the precondition of scale free network distribution, and the Topological Overlap Matrix (TOM) was constructed for clustering and segmenting the modules. The relationship between each network module and the sample phenotype were analyzed, so as to select the modules related to the time points.

### 2.4. Identification and Analysis of Vital Modules and Key Genes

The significance of the different time point modules was compared, with *p* < 0.05 indicating a statistically significant difference and a correlation of module–trait value *cor* ≥ 0.5 which indicating a vital module. To directly describe the gene expression in each vital module, we used singular value decomposition in transforming the gene expression data from the gene space to eigengene space, where eigengenes are the unique orthonormal superpositions of the genes for each module.

The module eigengene (MM) value can be obtained by analyzing the correlation between the expression of the gene and the corresponding module eigengene. The MM value is essentially a correlation coefficient. If the absolute value of MM is close to 1, it indicates that the gene is highly correlated with the module. The correlation analysis is performed between the expression of the gene and the corresponding phenotype value. The final value of the correlation coefficient is gene significance (GS). GS reflects the correlation between the gene expression and the phenotype data. The higher the GS, the more relevant the gene in the research phenotype. In this paper, we took MM ≥ 0.75 and GS ≥ 0.2 as the criteria to screen the key genes in the vital modules.

### 2.5. Function Enrichment and Visualization of Key Genes

The key genes found in two genotypes were subject to the Gene Ontology (GO) database and the Kyoto Encyclopedia of Genes and Genomes (KEGG) for function and pathway enrichment analysis; *p* < 0.05 was the significance threshold. The cluster and visualization of the key genes were constructed through the MCODE, Centiscape and Protein–Protein Interaction Networks (PPI) plugins of Cytoscape (3.9.0).

### 2.6. Selection of Candidate Hub Genes

A more comprehensive search for genes with high connectivity in both genotypes was carried out. The terms “heat stress” and “high temperature” were used to screen the related genes studied through the Oryzabase (https://shigen.nig.ac.jp/rice/oryzabase/gene/list, accessed on 1 March 2022) and RiceData (https://ricedata.cn/gene/, accessed on 1 March 2022). Combined with a clustering algorithm, high connectivity genes were defined as known–core genes. The known–core genes were compared with the key genes, and the candidate hub genes of the two genotypes were obtained by K-means clustering.

## 3. Results

### 3.1. Data Processing

Based on the DEGs at different time points across all of the samples, 20,631 and 20,742 genes were identified in the two genotypes, respectively (Supplementary Table S2). These genes were used for further WGCNA analysis after normalization.

### 3.2. Construction of Co–Expression Network by WGCNA

**Figure 1:**
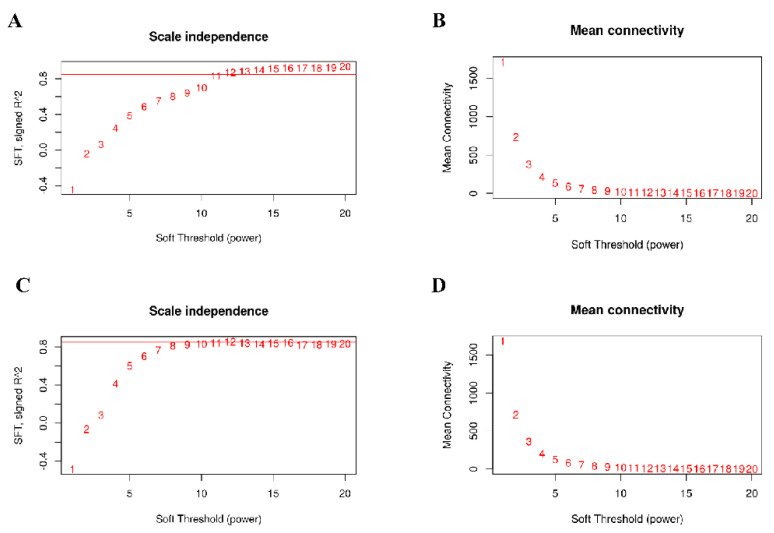
Network topology for different soft–thresholding powers of IR64 (**A**,**B**) and Koshihikari (**C**,**D**). The *x*-axis represents the weight parameter β. The *y*-axis of the left figure represents the square of the correlation coefficient between log(k) and log(p(k)) in the corresponding network. The *y*-axis of the right graph represents the mean of all gene adjacency functions in the corresponding gene module. The approximate scale free topology can be attained at the soft thresholding power of 12 in the two genotypes.

**Figure 2:**
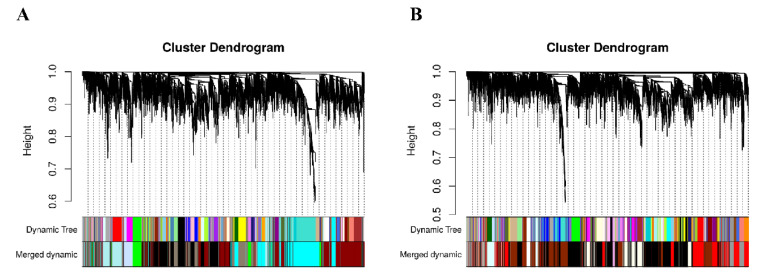
Gene modules identified by weighted gene co-expression network analysis (WGCNA) of IR64 (**A**) and Koshihikari (**B**). Gene dendrogram obtained by clustering the dissimilarity based on consensus Topological Overlap with the corresponding module colors indicated by the color row. Each colored row represents a color coded module, which contains a group of highly connected genes.

An appropriate soft threshold can effectively reduce the correlation noise in the adjacency matrix, which makes the network conform to the power law distribution and produce a higher similarity with a scale free network. In this study, the fitting degree of the scale free topological model was 0.85 and the soft threshold for network construction was selected as 12 for both genotypes (Figure 1). Hierarchical clustering was used to produce a hierarchical clustering tree of genes with branches and leaves, which represent the modules and genes, respectively. After determining the gene module according to the dynamic cutting method, the eigenvector value of each module was calculated in turn, and then the modules were analyzed in order to merge the modules that were close to each other into new modules. The obvious modules were identified and different colors were used to represent them (Figure 2).

**Figure 3:**
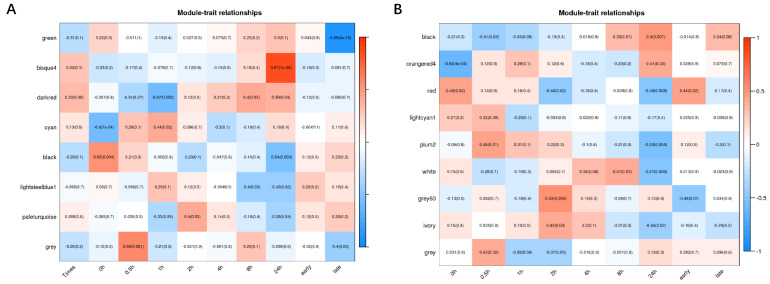
The correlation coefficient and correlation significance between the module and different time points of HS in IR64 (**A**) and Koshihikari (**B**). Each row in the table corresponds to a consensus module, and each column to a time point. The module name is shown on the *y*-axis, and the time point is shown on the *x*-axis. The table is color coded by correlation according to the color legend. Intensity and direction of correlations are indicated on the right side of the heatmap (red, positively correlated; blue, negatively correlated).

A total of eight modules including green (314 DEGs), bisque4 (710 DEGs), darkred (1602 DEGs), cyan (1233 DEGs), black (1195 DEGs), lightsteelblue1 (231 DEGs), paleturquoise (444 DEGs) and grey (83 DEGs) were obtained in IR64 (Figure 3A). Nine modules including black (2284 DEGs), orangered4 (1165 DEGs), red (975 DEGs), lightcyan1 (240 DEGs), plum2 (44 DEGs), white (97 DEGs), grey60 (271 DEGs), ivory (612 DEGs) and grey (124 DEGs) were obtained in Koshihikari (Figure 3B). These modules were positively or negatively correlated with different time points, and the genes in the corresponding modules were upregulated or downregulated, indicating that the genes respond to HS differently at different time points.

### 3.3. Modules Associated with Differences between IR64 and Koshihikari after HS

The preliminary study (unpublished data) shows that DEGs at 4 h post HS between the two cultivars were the lowest at all time points, suggesting that 4 h after HS might be an important critical time point. Thus, we added early (before 4 h) and late (after 4 h) time points to process (Figure 3A,B). Through the correlation analysis of module and time, with the absolute value of *cor* ≥ 0.5 and *p* < 0.05 as the criterion, we found several specific modules with significant correlations for IR64 and Koshihikari. For IR64, the darkred module (*r* = −0.57, *p* = 0.002) at 1 h, the bisque4 module (*r* = 0.87, *p* = 1 × 10^−9^) at 24 h, the black module (*r* = −0.54, *p* = 0.003) at 24 h and the green module (*r* = −0.89, *p* = 4 × 10^−10^) at the late time point showed high correlation with HS (Figure 3A, Table S3). For Koshihikari, the grey60 module (*r* = 0.53, *p* = 0.004) at 2 h, the black module (*r* = 0.5, *p* = 0.007) at 24 h, the plum2 module (*r* = −0.53, *p* = 0.004) at 24 h and the white module (*r* = −0.51, *p* = 0.006) showed high correlations with HS (Figure 3B, Table S4).

The correlation between each gene and eigengene was calculated, the module membership (MM) < 0.75, the gene significance (GS) < 0.2 of genes in the module were deleted, and we finally obtained key genes in the specific module (Tables S3 and S4). To further identify the features of the modules in response to HS, the key genes were analyzed through GO and KEGG. For IR64, the spliceosome, RNA transport, RNA degradation and ribosome biogenesis in eukaryotes were significantly enriched at the darkred module. The nucleic acid binding, zinc ion binding, biosynthesis of co–factors, spliceosome, oxidative phosphorylation and porphyrin and chlorophyll metabolism were significantly enriched at the bisque4 module. The metabolic pathways, biosynthesis of secondary metabolites and pathways related to photosynthesis, such as carbon metabolism and porphyrin/chlorophyll metabolism, were significantly enriched at the black and green modules. In addition, secondary metabolic pathways associated with resistance were also found at the 24 h time point, such as pyruvate metabolism, glyoxylate and dicarboxylate metabolism, butanoate metabolism and biotin metabolism (Figure S1A–D).

For Koshihikari, the grey60 module displayed significant enrichment in ribosome and oxidative phosphorylation. In terms of GO, ATP metabolic process, proton–transporting ATPase activity, rotational mechanism, translation elongation factor activity and cytochrome–c oxidase activity were found. For the plum2 module, protein processing in the endoplasmic reticulum and plant–pathogen interaction on KEGG terms, and small GTPase–mediated signal transduction and GTP binding on GO terms were found. For the black module, the highly enriched genes, mostly on DNA or RNA processes and repair pathways such as spliceosome, RNA transport, RNA degradation, nucleotide excision repair and mRNA surveillance pathway were found. At the white module, the biosynthesis of secondary metabolites, carbon metabolism, citrate cycle (TCA cycle) and carbon fixation in photosynthetic organisms were significantly enriched (Figure S2A–D). Based on the differences in the enrichment of module genes between the two genotypes, it can be considered that energy and secondary metabolism are the main resistance responses involved in HS, and the degradation and repair of Koshihikari’s genetic material may be the inherent manifestation of its poor heat tolerance.

### 3.4. Classification and Analysis of HS–Related Known–Genes

Through the Oryzabase and RiceData databases, we obtained 490 genes related to the HS of rice (Table S5). According to the density of the neighbor nodes, 490 genes were divided into 14 clusters (Figure S3) with 146 known–core genes (Table S5). These known–core genes included seven WRKY protein genes (*OsWRKY1, OsWRKY53, OsMYB30, OsWRKY24, OsWRKY28, OsWRKY10, OsMYB55*), six heat–shock transcription factor (HSF) genes (*OsHsfA4a, OsHsfA1, OsHsfA3, OsHsfA9, OsHsfB1, OsHsfA5*), a bHLH protein gene (*EAT1*) and a NAC protein gene (*LOC_Os11g03370*). In addition, 28 ribosomal protein–related genes (*LOC_Os12g38000, LOC_Os11g05370, LOC_Os01g04730, LOC_Os01g24690, LOC_Os01g62350, LOC_Os01g67134, LOC_Os02g18380, LOC_Os02g40880, LOC_Os03g37970, LOC_Os03g58204, LOC_Os04g39700, LOC_Os04g50990, LOC_Os05g11710, LOC_Os05g48220, LOC_Os05g49030, LOC_Os06g21480, LOC_Os07g01870, LOC_Os07g26740, LOC_Os07g44230, LOC_Os09g08430, LOC_Os09g24690, UbL401, LOC_Os09g32532, LOC_Os10g32820, LOC_Os11g24610, LOC_Os03g10930, LOC_Os02g10540, LOC_Os07g3609*0) and 10 heat–shock protein (HSPs) genes (*OsHSP1, hsp82B, OsHSP58.7, HSP70, HSP40, hsp82A, OsHSP74.8, OsHSP71.1, OsHSP26.7, Oshsp18.0–CII*) were regulatory nodes for HS.

### 3.5. Candidate–Hub Gene Analysis of IR64 and Koshihikari

**Figure 4:**
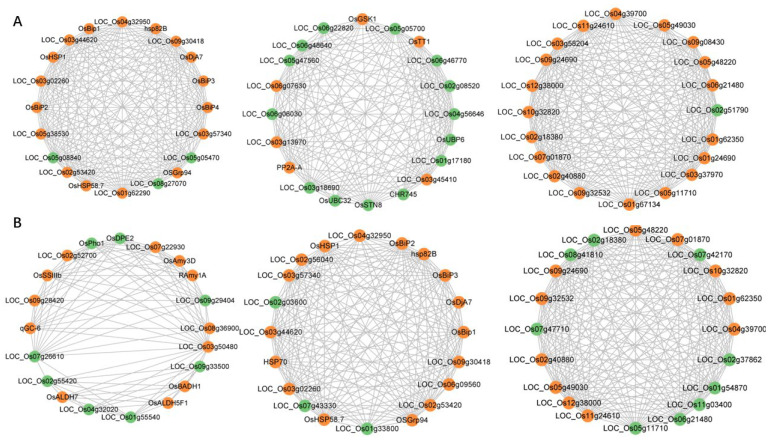
60 candidate–hub genes of IR64 (**A**) and Koshihikari (**B**) obtained from the interaction network analysis with known–core genes. Yellow color represents the genes from known–core genes; green color represents the genes from DEGs in this study.

In order to more effectively search for the hub genes’ response to HS, we performed an interaction network analysis of IR64 and Koshihikari key genes with known-core genes, respectively. According to the K-means clustering algorithm, the interaction network was divided into three main modes, and the top 20 genes of each mode were taken as candidate hub genes (Table S6). As shown Figure 4A,B, IR64 and Koshihikari obtained 60 candidate–hub genes, respectively, in response to HS. In the candidate–hub genes, for IR64, 16 genes had been cloned (*CHR745, hsp82B, OsBip1, OsBip2, OsBiP3, OsBiP4, OsDjA7, OsGrp94, OsGSK1, OsHSP1, OsHSP58.7, OsSTN8, OsTT1, OsUBC32, OsUBP6* and *PP2A–A*), with 19 putative ribosomal proteins and five putative Dnak family proteins included. For Koshihikari, 18 genes were cloned (*HSP70, hsp82B, OsALDH5F1, OsALDH7, OsAmy3D, OsBADH1, OsBip1, OsBip2, OsBip3, OsDjA7, OsDPE2, OsGrp94, OsHSP1, OsHSP58.7, OsPho1, OsSSlllb, qGC-6* and *RAmy1A*), along with 20 putative ribosomal proteins and two putative Dnak family proteins. Among the candidate–hub genes of IR64 and Koshihikari, 28 genes were identical, which implies that there was a consistent HS response among the genotypes. Overall, two genotypes of rice were found to share some of the same hub nodes for the response to HS; however, different specific regulation responses were also involved.

**Figure 5:**
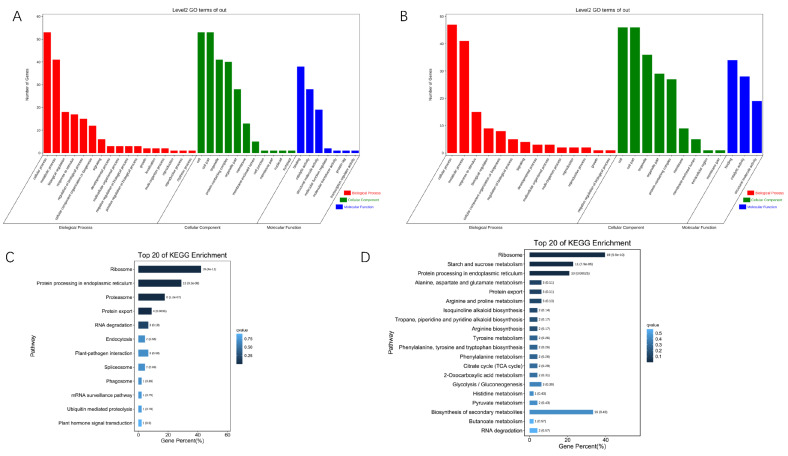
GO annotations (**A**,**B**) and KEGG enrichment (**C**,**D**) analysis of candidate–hub genes of IR64 (**A**,**C**) and Koshihikari (**B**,**D**). The *x*-axis of GO annotation shows GO terms at BP, CC and MF. The *y*-axis shows the number of genes correlating to the GO terms. The *x*-axis of the KEGG enrichment shows the gene percent of KEGG terms. The *y*-axis shows the KEGG enrichment terms.

To identify features of the 60 candidate hub genes for IR64 or Koshihikari in response to HS, function and pathway enrichments were performed. The GO enrichment results show that the candidate hub genes of IR64 and Koshihikari are enriched to consistent terms. Within BP, “cellular process” and “metabolic process” were the prominent enrichment terms. Within CC, “cell”, “cell part”, “organelle” and “organelle part” were the prominent enrichment terms. Within MF, “binding”, “structural molecule activity” and “catalytic activity” were the prominent enrichment terms (Figure 5A,B). In the KEGG enrichment metabolic pathway, ribosome and protein processing in the endoplasmic reticulum were common metabolic pathways, with more genes enriched in the two genotypes (Figure 5C,D). Unlike IR64, Koshihikari was more enriched in starch and sucrose metabolism and the biosynthesis of secondary metabolites pathways, suggesting that both pathways had important effects on the phenotype of Koshihikari under HS.

## 4. Discussion

Global warming is a serious problem. The high frequency and prolonged periods of HS have become a direct threat to the productivity of agricultural crops, including rice [24,25]. Under such severe conditions, the diverse genetic background of rice germplasms was selected and the molecular mechanisms were studied to cope with the effects of HS. However, due to the complexity of phenotypes under HS, relying on traditional map–based cloning to analyze the molecular mechanism of HS requires a significant amount of labor power and material resources. In recent years, the combination analysis of RNA–seq and WGCNA has already become a critical, cost effective method to discover the key genes and interactions that might be functionally related to stress [26]. The main purpose of this study was to reveal the different molecular mechanisms of the two genotypes, and to identify the hub genes in response to HS. By searching, sorting and clustering the studied heat stress related genes, the obtained known–core genes were used as the basis for finding the hub genes of two genotypes. To our knowledge, no cluster analysis of these HS genes has been conducted and applied to search for HS hub genes of different genotypes to date. Therefore, this work combines the RNA–seq data of the two genotypes with the studied HS genes, which helps comprehensively obtain the hub gene sets and identify important metabolic response pathways.

Through the correlation analysis of modules using time points and eigengenes, four modules were selected each for IR64 and Koshihikari. We took the highly correlated genes in the modules as core genes that were used for further biological function and metabolism analysis. In the four modules of the two genotypes, we found that carbohydrate metabolism, energy metabolism and amino acid metabolism were the elements of the metabolic pathways most involved in HS. Carbohydrate metabolism includes starch and sucrose metabolism, amino sugar and nucleotide sugar metabolism, and glyoxylate and dicarboxylate metabolism, among others [27]. Carbohydrate metabolism affects energy and carbon skeleton composition, which determine the survival strategy of plants under environmental stress [28]. Carbon availability is closely related to stress resistance. HS affects the activity of various carbon metabolizing enzymes, such as cell wall invertase (CWIN), ADP–glucose pyrophosphorylase (AGPase) and sucrose synthase (SuSy) [29,30,31]. Carbohydrate metabolizing enzyme activities could alter the carbohydrate biosynthetic pathway mechanism [32]. Due to changes in photosynthetic carbon metabolism, HS inhibits plant development, disrupts mineral–nutrient relationships, and impairs various types of physiological metabolism [33]. Moderate HS could induce a decrease in photosystem II (PSII) abundance, an increase in photosystem I (PSI), the reduction of plastoquinone and cyclic electron flow resulting in an increase in H_2_O_2_ and irreversible damage to the photosynthetic apparatus [34,35,36]. Most cellular energy is produced through oxidative phosphorylation in mitochondria. The first step in the pathophysiology of HS appears to be an increase in cellular energy demand, which relates to the increase of ROS [37]. Long term exposure to high temperatures leads to accelerated senescence, manifested as loss of chlorophyll and adjustment in amino acid metabolism [38]. Overall, the transcription level of genes related to energy production, utilization and antioxidant defense were significantly altered under HS, suggesting that the mechanisms combine with multiple pathways in response to HS.

A large number of HS–related QTLs have been identified and cloned, but efficient aggregation and clustering is lacking. In this study, we obtained 490 genes related to HS, and divided them into 14 clusters with 145 known–core genes. In total, 14 transcription factors (TFs), 28 ribosomal proteins (RPs), and 16 heat shock protein (HSPs) and heat–shock transcription factor (HSF) genes had a strong degree of association in the network of interaction. TFs play a regulatory function through the forming of transcriptional complexes, which bind to local and distal *cis*–elements of a given gene in a specific biological environment, affecting the expression of a vast number of downstream genes [39,40]. When plants are subjected to abiotic stresses such as drought, salt and HS, the TFs such as MYB, MYC, NAC, bZIP bind to the *cis*–elements MYBRS, MYCRS and ABRE (core sequence ACGTGGC) in the promoter region of downstream stress response genes *RD22*, *Gly*, *RD29B* and *RD20A* [41]. *NTL4*, an NAC transcription factor gene, forms a positive feedback loop with ROS, causing a sharp increase in ROS at high temperatures, recycling nutrients and metabolites from damaged tissues to the meristem or newly formed leaves, causing local programmed cell death (PCD), thereby enhancing plant survival. The constitutive overexpression of *AtWRKY25* and *AtWRKY26* could enhance the resistance to HS, as the reaction pathways are intertwined with the interactions of many plant hormones, calcium and ROS [42]. RPs are a conserved family in biological evolution, and are well known for their role in mediating protein synthesis and maintaining the stability of the ribosomal complex. The study of 34 candidate RPs genes showed that all of the RPs were highly responsive to stress, including heat, H_2_O_2_ treatments, salt and infection with a bacterial pathogen, xanthomonas oryzae, which causes leaf blight [43]. Various abiotic stresses could reduce ribosomal protein levels, resulting in the limits for ribosomal assembly rates and protein synthesis, which was the reason for RPs being located at the core nodes in the PPI network. HSPs have the function of a molecular chaperone and, under stress conditions, are thought to eliminate potentially harmful proteins arising from misfolding, denaturation or aggregation, form complexes with unfolded proteins, assist transmembrane transfer, and play an important role in stabilizing polypeptide chains and preventing protein inactivation, which contributes to cellular homeostasis in cells under HS [44,45]. As molecular chaperones, *HSPs* are activated to play protective roles when plants are confronted with various adversities [46]. An increase in the abundance of HSPs was universally observed in rice leaves under HS and some HSPs were assembled into a large hetero–oligomeric complex in response to HS [44]. The expression of HSPs is controlled and regulated by HSFs, which bind to *cis*–acting regulatory elements in the promoter region of the HSP genes; the activity of HSFs is also regulated via a feedback loop formed by the physical interaction with HSPs [47]. It has been proven that a number of HSFs and HSPs genes are the central regulators of the HS regulation and response network, which could activate the expression of many downstream genes [48,49,50]. Understanding the mechanisms of core TFs, RPs, HSPs and HSFs involved in HS will not only be a summary of the previous research on the core network map of HS, but will also provide a theoretical basis for the mining of new HS hub genes and field applications.

Core genes are the markers for the processes involved in a specific biological response, and hub genes usually play an essential role in gene regulation networks [51]. Based on the responses of the 145 known–core genes to HS, 60 highly connected genes were obtained for the two genotypes, respectively. A number of HSP and RP genes were found in the two genotypes, which proves their central position in the response to HS. In addition, some interesting genes were also found in the candidate hub genes. *OsDjA7* and *OsGrp94* were common genes in both genotypes. *OsDjA7*, the first characterized DnaJ gene, is involved in DNA replication and repair through the interaction with the proliferating cell nuclear antigen (PCNA) gene, and also functions in the chloroplast development of rice [52,53,54,55]. *OsGrp94* belongs to the HSP90 family protein. HSP90/Gas2/HSP40 could form a caspase–3–related protein complex in rice suspension cells’ response to HS. HSP90/Rac1/RAR1/HSP70 could form one or more protein complexes in rice cells and play important roles in the innate immunity of rice [56,57]. *OsDjA7* and *OsGrp94* not only respond to stress, but also function in slowing down or repairing the physiological damage caused by stress. *OsGSK1* and *OsTT1* were the unique response genes in IR64. *OsGSK1* has physiological roles in brassinosteroid (BR) signal transduction pathways and functions in several stress responses, including cold, heat, salt and drought [58,59]. Thermo–tolerance 1 (TT1), which encodes an α2 subunit of the 26S proteasome, is involved in the degradation of ubiquitinated proteins [60]. During the evolution of Asian rice into tropical japonica rice, temperate japonica rice and indica rice, *OsTT1* was obviously selected by environmental temperature, and then functional variation occurred to make the corresponding varieties adapt to their growth environmental temperature. The study of *Thermo-Tolerance1* (*OgTT1*), cloned from African rice (*Oryza glaberrima*), shows that *OgTT1* protects cells from HS through the more efficient elimination of cytotoxic denatured proteins and more effective maintenance of heat–response processes than achieved with *OsTT1* [60]. Two acetaldehyde dehydrogenase (ALDH) genes, *OsALDH5F1* and *OsALDH7*, and the betaine aldehyde dehydrogenase gene, *OsBADH1*, were the unique response genes in Koshihikari. ALDH is considered the antidote to reactive oxygen species in organisms. It oxidizes toxic aldehydes into corresponding non–toxic carboxylic acids, maintains the balance of aldehydes and plays an important role in stress physiology [61]. *OsBADH1*, encoding a key enzyme for the glycine betaine biosynthesis pathway, has a physiological function in the oxidation of acetaldehyde produced by catalase, which is involved in multifunctional mechanisms in response to environmental stresses, including salt, plasmolysis, temperature and light stress [62,63,64].

The GO and KEGG enrichment analyses of the 60 candidate–hub genes unveiled an interesting result in the two genotypes. IR64 and Koshihikari had consistent GO terms and partly common metabolic pathways. However, starch and sucrose metabolism and the biosynthesis of secondary metabolite pathways were the unique metabolic pathways. Starch and sucrose metabolism is the downstream branch of carbohydrate metabolism. When photosynthesis is damaged, starch and sucrose do not only provide carbon energy, but also behave as osmo protectants and compatible solutes to alleviate the negative effects of stress [65]. Secondary metabolism refers to the process of the biosynthesis of non–essential substances and the storage of secondary metabolites [66]. The plant secondary metabolic process is the result of plants’ adaptation to the ecological environment over long–term evolution, and it plays an important role in dealing with the relationship between plants and the ecological environment [67,68,69]. Constitutive high molecular–weight secondary metabolites such as lignin and melanin can act as physical barriers to pathogen invasion [70]. The plant hormones jasmonic acid and salicylic acid are involved in signaling molecules for disease resistance. Proline, sugar alkaloid, abscisic acid and soluble sugar are used as osmotic substances that participate in alleviating various abiotic stresses, such as high temperature, drought and salinity [71]. The different metabolic pathways of the candidate hub genes between the two genotypes reflect the inconsistency in response to HS. Starch and sucrose metabolism and the biosynthesis of secondary metabolites might play a critical role in the sensitivity of Koshihikari to HS. Moreover, it should not be ignored that there were many expressed proteins with unknown functions in the candidate hub genes of the two genotypes in this study; thus, further research is still needed, as these genes may have important functions in coping with HS.

## 5. Conclusions

In summary, a gene co–expression network based on WGCNA was constructed using the transcriptome scale changes of the two genotypes under HS. The highlight of this study is that the core genes were systematically screened based on the studied HS research. Through the co–expression modules identified at different time points and the interaction network analysis with known HS related genes, we took the top 20 genes in terms of their degree of connection as candidate hub genes. The analysis of these candidate hub genes found that, compared with IR64, starch and sucrose metabolism and the biosynthesis of secondary metabolites were more significantly enriched in Koshihikari, indicating that starch and sucrose metabolism and the biosynthesis of secondary metabolites might play a critical role in response to HS for Koshihikari. Even though the results have some limitations and further experimental studies are required to verify the function in response to HS, a reliable basis remains for further research into the molecular mechanisms and the mining of key genes in response to HS.

## Data Availability

The data presented in this study are available upon request from the corresponding author.

## Supplementary Materials

The following supporting information can be downloaded at: https://www.mdpi.com/article/10.3390/genes13061020/s1. Table S1: DEGs in IR64 and Koshihikari at different time points. Table S2: DEGs in both genotypes at all the time points. Table S3: Key genes of different modules in IR64. Table S4: Key genes of different modules in Koshihikari. Table S5: Analysis of known–genes related to HS of rice obtained from Oryzabase and RiceData databases. Table S6: Candidate hub genes of IR64 and Koshihikari. Figure S1: Analysis of modules in IR64 through GO and KEGG. Figure S2: Analysis of modules in Koshihikari through GO and KEGG. Figure S3: Cluster of heat stress related genes.
